# Environmental impacts of household consumption in Europe: Comparing process-based LCA and environmentally extended input-output analysis

**DOI:** 10.1016/j.jclepro.2019.117966

**Published:** 2019-12-10

**Authors:** Valentina Castellani, Antoine Beylot, Serenella Sala

**Affiliations:** European Commission-Joint Research Centre, Via Enrico Fermi 2749, I-21027, Ispra, VA, Italy

**Keywords:** Life cycle assessment, Consumer footprint, Basket of products, Input-output analysis, EXIOBASE 3, Household consumption

## Abstract

The environmental impacts generated by household consumption are generally calculated through footprints, allocating the supply-chain impacts to the final consumers. This study compares the result of the Consumer Footprint indicator, aimed at assessing the impacts of household consumption in Europe, calculated with the two standard approaches usually implemented for footprint calculations: (i) a bottom-up approach, based on process-Life cycle assessment of a set of products and services representing household consumption, and (ii) a top-down approach, based on environmentally extended input-output tables (EXIOBASE 3). Environmental impacts are calculated considering 14 environmental impact categories out of the 16 included in the EF2017 impact assessment method. Both footprints show similar total values regarding climate change, freshwater eutrophication and fossil resource use, but in the meantime very large differences (more than a factor 2) regarding particulate matter, photochemical ozone formation, land use and mineral resource use. The exclusion of services in the bottom-up approach can explain only to some extent these differences. However, the two approaches converge in identifying food as the main driver of impact in most of the impact categories considered (with a generally lower contribution in top-down compared to bottom-up). Housing and mobility are relevant as well for some impact categories (e.g. particulate matter and fossil resource depletion). Some substances are identified as hotspot by both approaches, e.g. the emission of NH_3_ to air (for acidification and terrestrial eutrophication), of NOx to air (for acidification, marine and terrestrial eutrophication, and, to some extent, photochemical ozone formation), of P to water and to soil (for freshwater eutrophication) and of fossil CO_2_ to air (for climate change). Significant differences at the inventory side are key drivers for the differences in total impacts. These include: (*i*) differences in the intensity of emissions, (*ii*) differences in the coverage of elementary flows, (*iii*) differences in the level of detail relative to elementary flows. Overall, the key converging results from both approaches (in particular regarding most contributing areas of consumption and substances) can be considered as a robust basis to support the definition of policies aimed at reducing the environmental footprint of household consumption in Europe.

## Highlights

• EU Consumer Footprint is assessed comparing a bottom-up and a top-down approach.
• Environmental impacts calculated with the two approaches are different in absolute terms.
• The two approaches converge in identifying food consumption as a key driver of environmental impacts.
• Housing and mobility are relevant as well for some impact categories.
• A specif assesment of the relevance of the use phase over the life cycle impacts is conducted.

## 1 Introduction

The Agenda 2030 is currently one of the global key reference towards sustainable development, setting 17 Sustainable Development Goals (SDGs) (UN, 2015; EC, 2016). Responsible consumption and production are the focus of the SDG12, while being also addressed by other SDGs (e.g. SDG 11 on sustainable cities and communities, the SDG 8 on economic growth). Furthermore, as consumption directly and indirectly (along the supply-chain) induces environmental impacts, it additionally affects several other SDGs (e.g. the SDG 13 on climate action). In this context, a number of approaches has been developed over time to assess impact of consumption, both at micro and macro scale. Life cycle thinking and assessment have emerged as key approaches to pave the way towards Sustainable Consumption and Production. One specific field of research is related to the assessment of the sustainability of household consumption. So far, the majority of studies have focused on specific consumption domains (e.g. energy or waste), with only limited use of environmental indicators in the assessment (Caeiro et al., 2012). In this context, the development of indicators such as the Household Sustainable Consumption Index (Bartolj et al., 2018), based on variables, appears promising to monitor and encourage any progress towards sustainable consumption.

Impacts of household consumption are generally calculated by means of footprints, often pressure-based. Some examples are the carbon footprint (Hertwich and Peters, 2009; Davis and Caldeira, 2010), the water footprint (Hoekstra and Mekonnen, 2012), the land footprint (Bruckner et al., 2015), the material footprint (Wiedmann et al., 2013), etc. All those indicators adopt a consumption-based approach, i.e. they consider the full life cycle of products and they allocate the impacts to the final consumer (differently from a production-based approach, which allocates the impacts to the producer of goods).

On the one hand, several studies implement a top-down approach, using Environmentally Extended - Multi Regional Input Output (EE-MRIO) tables to assess the environmental impacts of final consumption. Top-down methods have the advantage of providing a consistent framework for the allocation of environmental burdens from the overall emissions and resources consumption generated by economic systems at macro scale to the expenditures of final consumers. However, the top-down methods lack details at the product level. The level of aggregation of products and services in EE-MRIO tables is generally much larger than the product level considered in standard process-based life cycle assessment (LCA) studies (for example, 200 products and 163 industries are distinguished in the EE-MRIO tables of EXIOBASE 3; Stadler et al., 2018).

On the other hand, other studies follow a bottom-up approach, based on the LCA of representative products, which are then up-scaled to overall consumption figures through several up-scaling techniques (e.g. EC-JRC, 2012, Sala and Castellani, 2019). Some LCA-based studies aim at monitoring the environmental impacts of household consumption at national scale, either focusing on specific footprints (e.g. carbon footprint by ADEME, 2012, for France), or considering a wider set of LCA-based indicators (e.g. Kalbar et al., 2016, for Denmark). Other studies primarily focus on a specific area of consumption in a geographical region: e.g. Teubler et al. (2018), on household goods in Germany, Eberle and Fels (2016) on food consumption in Germany, Lavers et al. (2017) on overall consumption in urban areas in four Swedish cities, and Ding et al., 2019 on the greenhouse gas emissions of Chinese urban household consumption. Thanks to the use of representative products with detailed life cycle inventories, the bottom-up methods hold a more realistic picture and a high level of detail for what concerns specific products, and they are more useful when modelling scenarios acting on specific features of single products or on user behaviour. On the contrary, the use of representative products may reduce the representativeness of the model with reference to the total amount of impact generated, because it implies the exclusion of products that are less relevant in terms of the amount consumed.

Acknowledging that both approaches have strengths and weaknesses, the present study compares the results of the application of top-down and bottom-up approaches for the assessment of the environmental impacts of household consumption in Europe (called here Consumer Footprint). The aim of the study is twofold: firstly, to identify converging results from the two approaches, in support to the definition of policies aimed at reducing those impacts. For this purpose, this study implements a common characterization framework in both modelling approaches, enabling to go beyond the limited set of impact indicators as often considered in existing studies. Moreover, it not only aims at identifying the most contributing areas of consumption, as usually considered, but it also assesses the relevance of the use phase, of services (disregarded in process-based LCA studies), and of the major substances contributing to the impacts. Secondly, this study identifies the methodological differences that lead to the different results in the two approaches, contributing to the discussion on key improvements needed in future studies aiming at the quantitative, life-cycle-based, assessment of the environmental impacts of household consumption.

## 2 Method

This study compares two modelling approaches, aimed at assessing the environmental impacts of household consumption in Europe in a life-cycle perspective. The two approaches are:

• the process-based LCA Consumer Footprint (called “pLCA-CF” in the following), which adopts a bottom-up approach and uses process-based LCA of representative products to calculate the corresponding LCI, as illustrated in Sala et al. (2019) and in Sala and Castellani, 2019, characterised with Environmental Footprint (EF) Life Cycle Impact Assessment (LCIA) method (EC, 2017)
• the Input-Output Consumer Footprint (called “IO-CF” in the following), which adopts a top-down approach, based on the inventory of resources and emissions as estimated using EXIOBASE 3 and characterised with the EF LCIA method as presented in Beylot et al. (2019a).

The geographical scope of the assessment is the European Union (EU) for both modelling approaches. However, the pLCA-CF refers to EU-27 whereas the IO-CF refers to EU-28 (i.e. is including Croatia). The study adopts a consumption-based approach. This implies to consider all the environmental impacts generated (either directly or indirectly) by the purchase and use of products and services by European citizens, i.e. including also impacts generated in the production of goods outside the European geographical boundaries. The reference year is different in the two modelling approaches, because of data constraints: the pLCA-CF is calculated using apparent consumption in EU-27 in 2010, whereas for the IO-CF the year 2011 is considered (expected to be the most up-to-date and reliable year among EXIOBASE 3 data series for any analysis at a disaggregated level; Schmidt, 2019). Despite results have also been calculated regarding year 2015 in the case of pLCA (Sala and Castellani, 2019), they cannot be derived for years more recent than 2011 with the hybrid version of EXIOBASE 3, and are therefore not further explored in the following comparison.

### 2.1 The process-based LCA Consumer Footprint (pLCA- CF)

For the calculation of the pLCA-CF, five areas of household consumption are considered: housing, mobility, food, household goods, and appliances. Each area of consumption entails a number of products (e.g. food products) and services (e.g. km travelled by car) consumed by households (see the Supporting information (SI) file for details on what is included in each area). Three of these areas (food, mobility, and housing) were identified in previous studies as the most relevant ones in terms of environmental impacts of consumption (e.g. Tukker et al., 2016). Two others were added to account for consumption of consumer goods in households and for energy-related devices and appliances. For each area, a process-based LCI model for a Basket of Products (BoP) that represent the most relevant product groups in the area considered was built. Representative products are selected by importance in mass and economic value, based on statistics on consumption and stock of products. The pLCA-CF in EU is calculated as the sum of the five BoPs. The life cycle phases considered for each basket are upstream activities (e.g. agricultural phase for the BoP Food and manufacture of product components for BoP Household goods), production, packaging, logistics, use phase, maintenance, and end of life (EoL). Those phases are adjusted to the specific features of each basket when needed (more details on what is included in each phase can be found in Table S1). When modelling production and logistics of consumer products, the world-wide dimension of the current supply chains has been considered, e.g. by modelling specific production conditions (as country-specific electricity mixes) based on the share of products that is imported to Europe from abroad and on the average distribution of the main countries of production, such as Pakistan and India for textiles (a detailed description of the assumptions made is given in Sala and Castellani, 2019).

The unit of analysis (functional unit) of the pLCA-CF approach is the household consumption in EU-27 in the five areas of consumption (reference year 2010). A description of the modelling approach undertaken in this study is provided in the following, while extensive details on data sources and modelling assumptions may be found in different specific studies, namely: BoP Housing (Baldassarri et al., 2017; Lavagna et al., 2018), BoP Mobility (Castellani et al., 2017a), BoP Food (Castellani et al., 2017b), BoP Appliances (Reale et al., 2019) and BoP Household goods (Castellani et al., 2019).

The LCIs of the representative products are in line with the International Life Cycle Data system (ILCD) guidelines (EC-JRC, 2010). The main data sources used for building the inventories are ecoinvent v.3.2 (BoPs housing, mobility, household goods and appliances) and Agrifootprint v.2 (BoP food) databases, complemented with data from the scientific literature.

In order to build the inventory of each BoP with reference to the functional unit considered (i.e. consumption in EU-27 in 2010), the LCIs obtained for each representative products are multiplied by the amount of that product that is consumed in one year by EU-27 citizens (Tables S2–S6).

To calculate the pLCA-CF in EU, the five BoPs are added together. When doing this, two issues have to be considered. Firstly, there are activities that may be part of the life cycle of more than one BoP (especially for the use phase). To avoid double counting when summing all BoPs, overlapping activities are included only once (in the BoP with the larger scope). This is done, for instance, for the electricity use: the electricity needed for using the appliances is put to zero in the BoP Appliances, and it is kept in BoP Housing, which includes the overall amount of electricity used in the European houses for several purposes, including appliances (the complete list of assumptions made to avoid double counting is reported in Sala and Castellani, 2019). Secondly, each BoP should take into account, to the maximum extent possible, the total consumption in the area under investigation. In fact, the representativeness of the products selected differs for the five BoPs. While BoP Housing is built to represent 100% of the EU building stock and BoP Mobility considers about 98% of the km travelled in Europe with private and public means of transport, the three other BoPs are instead built using representative products, subsequently not including 100% of the products consumed. Quantities of representative products in these three BoPs Food, Household goods and Appliances have been up-scaled to enlarge their representativeness. Hence, the final overall impact is the results of the following equation:

[1] pLCAConsumerFootprint=BoPHousing+BoPMobility+BoPFood×Upscalingfactor+BoPHouseholdGoods×Upscalingfactor+BoPAppliances×Upscalingfactor

For example, the three representative products under the product group “Meat” in the BoP Food (76.7 kg/person*yr^−1^ in total) represent 89% of the quantity of the overall meat purchased by European citizens in one year (86.4 kg/person*yr^−1^). Therefore, the quantity of the three meat products included in the basket is up-scaled to 100% (i.e. to 86.4 kg/person*yr^−1^) to represent the total amount of meat consumed (i.e. including also other types of meat, not modelled in the BoP).

It is important to remark that due to the upscale, the representativeness of BoP Food, BoP Appliances and BoP Household goods is 100% for the specific product groups considered. Still they do not cover 100% of food, appliances and household goods consumption, because there may be other product groups that are part of those areas of consumption and that are not included in the BoP.

### 2.2 The IO Consumer Footprint (IO-CF)

In the calculation of the IO-CF, the EEMRIO tables of EXIOBASE has been used for building the inventory, subsequently characterised with the Environmental Footprint LCIA method. Methodological details on the MRIO analysis and on the allocation of emissions and resources on COICOP categories are reported.

#### 2.2.1 Multi-regional Input-Output Analysis

Standard economic Input-Output Analysis is based on the Leontief inverse equation:

[2] $${x=\left(I-A\right)}^{-1}f$$

considering x the vector of output productions, A the technological requirement matrix and f the final demand. The inventory of emissions to the environment and of resources extracted from the environment, as a response to a given final demand, is derived according to:

[3] $$g=Bx=B{\left(I-A\right)}^{-1}f$$

with B the matrix of resources and emissions intensities per economic activity (that is, the coefficients of natural resources extraction and emissions per unit of output in the economic activities). In MRIO models, the standard IO matrices are extended: each industry in each region is differentiated considering separate rows and columns (Hertwich and Peters et al., 2010). In this study, Input-Output Analysis is implemented considering the hybrid version of EXIOBASE as the MRIO database supporting calculations (EXIOBASE 3.3.8, referred to as EXIOBASE 3 in the following; Stadler et al., 2018; Merciai and Schmidt, 2018). In EXIOBASE 3, MRIO Tables are available for 43 countries, including the 28 EU countries under focus in this study, plus 5 rest-of-world regions. The IO tables used for calculations integrate investments, based on the approach developed in the FORWAST project (Schmidt, 2010). Accordingly, both the IO-CF and the pLCA-CF account for capital goods.

The use phase in the IO-CF is calculated in an approach consistent with that of the process-based LCA. It includes direct emissions, electricity consumption, wastewater treatment and fossil fuels, steam and hot water consumed by households (see SI section 11 for a detailed description of products and services consumed by households and considered as part of the use phase in the IO-CF).

As observed in Beylot et al. (2019a), direct emissions of particulate matter (PM) PM_2.5_ and Non-Methane Volatile Organic Chemicals (NMVOCs) from households are underestimated in EXIOBASE 3.3.8, whereas these two substances have large contributions to the impact of EU final consumption on particulate matter and photochemical ozone formation. Hence, EXIOBASE 3.3.14 is used as the supporting database in this specific case of emissions, as in Beylot et al. (2019a). Household consumption in the 28 EU countries (f in equations [1], [2]), is drawn from EXIOBASE 3 considering year 2011 and differentiating 137 categories of products and services either in physical or monetary units. The IO-CF covers the entire consumption of households, whereas the pLCA-CF is limited to five areas of consumption. Only part of the consumption of services accounted for in the IO-CF are considered in the pLCA-CF (see SI file S10). Besides, in the pLCA-CF, the categories of products and services covered are modelled with a higher level of granularity (at the level of products instead of product categories).

#### 2.2.2 Allocation of emissions and resources to COICOP divisions

The direct implementation of EXIOBASE 3 enables to differentiate the contribution of 137 products and services to the total emissions and resource use induced by household consumption, in a nomenclature close to that of the Statistical Classification of Economic Activities in the European Community (NACE), with additional disaggregation considering specific products and services such as agriculture and food products, energy and waste treatment (Stadler et al., 2018). To facilitate the comparison between the two approaches implemented, the contribution of the 137 products and services to impacts in the IO-CF is further aggregated in a nomenclature of consumption (the Classification of individual consumption by purpose; COICOP) closer to the nomenclature of areas of consumption used for the pLCA-CF (see SI -S9, which details the allocation of EXIOBASE products and services to COICOP divisions enabling the calculation of emissions and resources per COICOP division).

Despite there is no one-to-one correspondence between the 5 BoPs and the 12 COICOP divisions, several of the latter correspond to the areas of consumption covered by the BoPs. The BoP Food covers the COICOP divisions “Food and non-alcoholic beverages”, “Alcoholic beverages, tobacco and narcotics” and “Restaurants and hotels”; the BoP Mobility covers “Transport”; the BoP Housing covers “Housing, water, electricity, gas and other fuels”; the BoPs Appliances and Household Goods cover 5 different COICOP divisions: “Miscellaneous goods and services”, “Furnishings, household equipment and routine household maintenance”, “Recreation and culture”, “Communications”, and “Clothing and Footwear” (more details on these correspondences in SI-S9).

Overall, the allocation approach of Huysman et al. (2016) was used as main reference, with complementary use of the ones implemented in Schmidt (2010) and Ivanova et al. (2017).

### 2.3 Impact assessment method

The inventories of household consumption in Europe (EU-27 for pLCA-CF and EU-28 for IO-CF), as obtained by the two approaches, were characterized with the EF LCIA method, package 2.0 (EC, 2017). The inventories have been mapped towards the EF nomenclature of elementary flows to allow the characterisation of the impacts. In total, 14 impact categories are considered: climate change (CC); acidification (AC); terrestrial eutrophication (TEU); marine eutrophication (MEU); freshwater eutrophication (FEU); particulate matter (PM); photochemical ozone formation (POF); human toxicity, cancer effects (HTOX–c); human toxicity, non-cancer effects (HTOX-nc); freshwater ecotoxicity (ECOTOX); land use (LU); water use (WU); minerals and metals resource use (MRD); and fossils resource use (FRD). In addition, two impact categories included in the original EF method (namely ozone depletion and ionising radiation) are not considered in the present study because EXIOBASE 3 does not include (or only very partly includes) the corresponding environmental extensions.

The EF2017 method includes characterization factors for 1402 elementary flows out of the 2020 present in the inventory of the pLCA-CF approach. Regarding the IO-CF, the inventory of emissions and resources includes a more limited set of elementary flows (78 in total: 36 mineral, metal and energy resources, 5 types of land occupation, 3 types of water consumption, 29 substances emitted to air, 2 to water and 3 to soil). In order to perform impact characterization, the mapping of characterization factors to each of the flows reported in the EXIOBASE environmental extensions has been performed, according to the systematic correspondence to the EF2017 nomenclature of Beylot et al. (2019a).

## 3 Results and discussion

Results of pLCA-CF and IO-CF are firstly compared in absolute terms, i.e. as total impacts generated by household consumption in Europe. Moreover, the drivers for these impacts (and differences between approaches) are explored, focusing on: contribution of services, hotspot analysis by areas of consumption and by substances, and finally contribution of the use phase and direct emissions. Results that are converging between the two approaches are considered more robust, e.g. in terms of drivers of impacts to be addressed by future policies.

### 3.1 Comparison in absolute terms

**Fig. 1:**
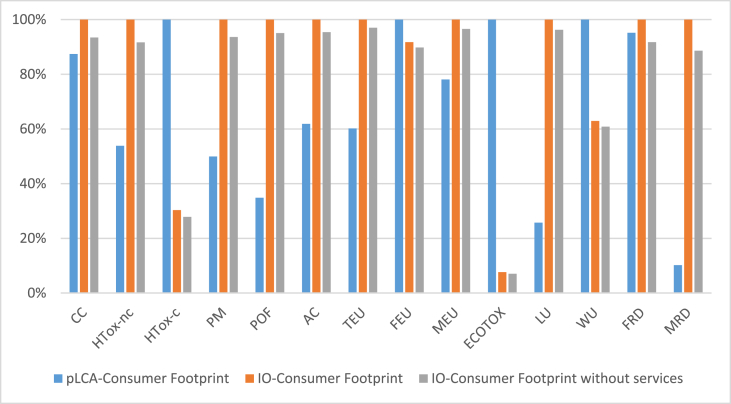
Comparison of pLCA-Consumer footprint and IO-Consumer footprint (the latter with and without services).

The characterized environmental impact of household consumption in Europe calculated with the two approaches is quite different in absolute terms (Table 1) and it is illustrated in percentage differences in Fig. 1. Three classes of impact categories can be identified. Firstly, the impacts due to CC, FEU, and FRD are comparable in the two approaches, showing less than 15% of difference. Moreover, regarding a second group of impact categories (HTOX-nc, PM, AC, TEU, MEU, and WU), the difference between IO-CF and pLCA-CF is relatively high but still below 60% of the highest value between the two approaches. Finally, regarding HTOX-c, POF, ECOTOX, LU, and MRD, the difference between the impacts calculated with the two approaches is more than 40% of the highest value between the two, and up to 90% or more in the case of LU and MRD. Furthermore, it can be observed that for ten impact categories (out of the 14 under study), the IO-CF is larger than the pLCA-CF.

**Table 1:** Characterized impact of household consumption in Europe according to the pLCA-Consumer Footprint and the IO-Consumer Footprint (either including or excluding the contribution of services).

Impact category	Code	Unit	pLCA-Consumer Footprint	IO-Consumer Footprint	IO-Consumer Footprint without services
Climate change	**CC**	kg CO_2_ eq	4.79E+12	5.48E+12	5.12E+12
Human toxicity, non-cancer	**HTox-nc**	CTUh	2.52E+05	4.68E+05	4.29E+05
Human toxicity, cancer	**HTox-c**	CTUh	7.15E+04	2.17E+04	1.99E+04
Particulate matter	**PM**	Disease incidence	3.61E+05	7.23E+05	6.77E+05
Photochemical ozone formation	**POF**	kg NMVOC eq	1.31E+10	3.76E+10	3.57E+10
Acidification	**AC**	molc H^+^ eq	3.63E+10	5.87E+10	5.60E+10
Eutrophication, terrestrial	**TEU**	molc N eq	1.21E+11	2.01E+11	1.95E+11
Eutrophication, freshwater	**FEU**	kg P eq	4.11E+08	3.77E+08	3.69E+08
Eutrophication, marine	**MEU**	kg N eq	1.14E+10	1.46E+10	1.41E+10
Ecotoxicity, freshwater	**ECOTOX**	CTUe	7.04E+12	5.37E+11	4.94E+11
Land use	**LU**	Pt	2.42E+14	9.40E+14	9.05E+14
Water use	**WU**	m^3^ water eq	6.64E+12	4.18E+12	4.04E+12
Resource use, fossils	**FRD**	MJ	6.10E+13	6.41E+13	5.88E+13
Resource use, mineral and metals	**MRD**	kg Sb eq	2.51E+07	2.46E+08	2.18E+08

There can be several reasons behind those differences, including:

− the scope of the assessment: the pLCA-CF is limited to the areas of consumption covered by the five BoPs and to the products included in those areas, whereas the IO-CF encompasses the whole household consumption;
− the uncertainty embodied in the inventory data considered in the two approaches, which propagates to the impact assessment results. In the top-down approach, uncertainty in particular lies in the construction of MRIOTs (uncertainty regarding mass and economic flows of products and services and environmental extensions) as well as in the level of aggregation of products and services (the full demand of European households is represented at a rather coarse level of details). In the bottom-up approach, uncertainty in particular lies in LCI data, for some of them based on a limited set of data sources completed by use of proxies and by considering representative products only;
− the year considered in the assessment: 2010 in the pLCA-CF, compared to 2011 in the IO-CF;
− the geographical scope considered: EU-27 in the pLCA-CF compared to EU-28 in the IO-CF;
− the number of substances (as elementary flows) considered in the two inventories (1402 in pLCA-CF versus 78 in IO-CF);
− the regionalisation of WU impacts (in pLCA-CF) or the specification of compartments and sub-compartments of emissions (which often imply that a different characterization factor is associated at the impact assessment stage to a specific sub-compartment).

The influence of cut-offs on services in the bottom-up approach, and of the different level of detail in the inventories (e.g. specification of emission compartments and inclusion/exclusion of some substances), are specifically scrutinized further in the following sections, as part of the hotspot analysis. On the contrary, this study disregards the other aspects listed, whose influence is expected to be only limited. Firstly, the inclusion of Croatia (the 28th EU country) in the IO-CF is responsible for no more than 1% of the difference with the pLCA- CF for most of the impact categories. The only exceptions are LU (3%), MRD (4%) and POF (5%). Moreover, the difference in the year is expected to have only limited effect on the results. In particular, the volume of household consumption in EU-28 only increased by 0.2% between 2010 and 2011 (Eurostat, 2018). Similarly, variations in the composition of household consumption from one year to another are expected to generate relatively limited effects on the results compared to the influence of the method undertaken, that is under focus in this study. Finally, the uncertainty embodied in the inventory data from ecoinvent database, used for the pLCA-CF, can be assessed using a pedigree matrix (Weidema et al., 2013) whereas, up to now, EXIOBASE 3 does not include any assessment of the uncertainty related to inventory data.

### 3.2 Contribution of services

**Fig. 2:**
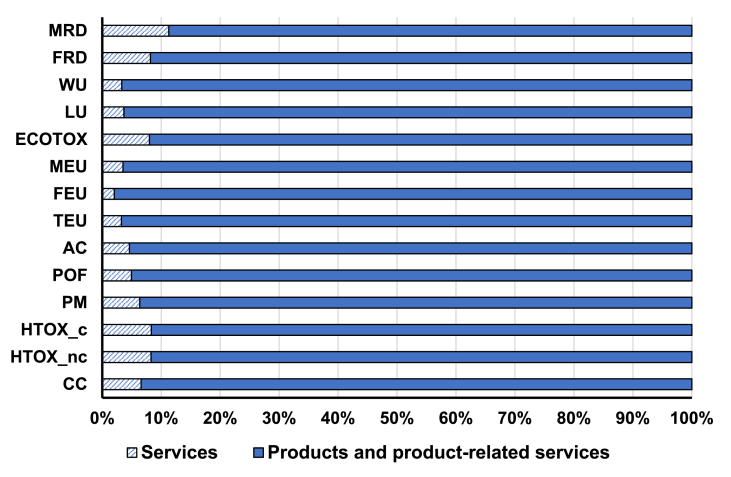
Contribution of “services” versus “products and product-related services” to the IO-Consumer Footprint (see SI S9 for the classification of “Services” and “Products and product-related services” in this study). The extended names of impact categories are reported in Table 1.

As shown in Fig. 2, the contribution of expenditures on services (e.g. "Recreational, cultural and sporting services"; "Health and social work services"; "Post and telecommunications") to the IO-CF is quite limited in almost all the impact categories (generally below 10%). Yet it has to be considered that the remaining impact is not totally related to the life cycle of products as calculated in the pLCA-CF, because the classification of activities in EXIOBASE nomenclature sometimes does not allow for a clear distinction between production activities and product-related services. For instance, the activity “Real estate services” (for which household expenditures are classified as “Products and product-related services” in Fig. 2) relates to a product (dwellings in the BoP housing) but also to service activities, in particular linked to the renting of dwellings. Moreover, in the top-down approach, impacts of services are accounted for not only as directly induced by household consumption, but also indirectly induced along the supply-chain of products and services consumed by households (see SI file S10, on the classification of services adopted for this study). In the end, the contribution of services directly consumed by households (as represented in Fig. 2) is to be considered as the lower bound of impacts induced by “services” that are excluded from the pLCA-CF approach.

The comparison of results of pLCA-CF and IO-CF without the impact of services (Table 1) shows that the exclusion of “services” in the bottom-up approach can explain only to some extent the difference in results from the top-down approach. In fact, FRD and CC are the only impact categories for which the impact calculated with top-down excluding services is close to the one calculated with the pLCA-CF (−4% and 7% difference, respectively).

### 3.3 Hotspot analysis, by areas of consumption

**Fig. 3:**
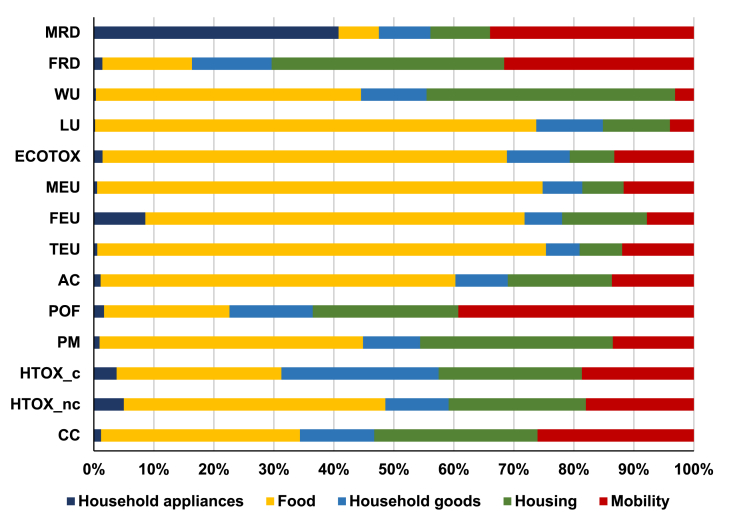
Contribution of the five BoPs to the total pLCA-Consumer Footprint in Europe, in 2010.

**Fig. 4:**
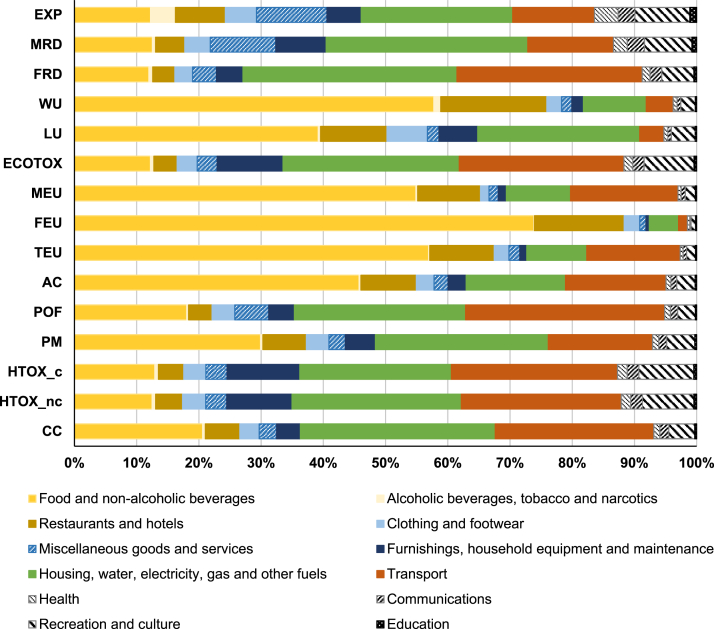
Contribution of COICOP divisions to the total IO-Consumer Footprint in Europe, in 2011. The contribution of the divisionsto the to total expenditures (EXP) is reported as well. The extended names of impact categories are reported in Table 1.

In the following paragraphs, the contribution of products and services is scrutinized based on the two approaches, in order to highlight converging results in terms of hotspots coming from household consumption in Europe and to identify possible sources of the discrepancies observed. Fig. 3 and Fig. 4 show the contribution by area of consumption according to the two approaches analysed, respectively considering the five BoPs (in the case of the pLCA-CF), and the 12 COICOP divisions (in the case of the IO-CF; see absolute results in Table S7). When interpreting the results, it has to be kept in mind that there is no one-to-one correspondence between the different types of areas of consumption from one approach to the other, by nature of the approaches implemented (see SI S9).

Impact assessment results show that food is the main driver for impacts on AC, TEU, FEU, MEU, PM, LU and WU in both Consumer Footprints, in particular contributing to more than 70% of the impact on TEU, MEU and LU for the pLCA-CF (Fig. 3, Fig. 4). Yet, the lower contribution of food sector in the IO-CF compared to the pLCA-CF is observed as a recurrent difference.

For the impact categories FEU and LU, the two approaches converge in the identification of food consumption as the main sector contributing to the impact (with a complementary significant contribution of Restaurants and Hotels in the top-down approach), but they diverge in the attribution of shares to the other sectors. Housing is the second most important sector for FEU in both cases, still with different contributions (about 15% in the pLCA-CF, compared to less than 10% in the IO-CF). Similarly, appliances and transport show a higher share in the pLCA-CF than in the IO-CF. The contribution from transport in the pLCA-CF comes mainly from the emission of Phosphate to water, related to the treatment of sulfidic tailings from mining of precious metals, which are used in printed circuit boards of cars. On the contrary, no emission of phosphorous to water from mining and mineral processing activities is accounted for in EXIOBASE 3. Conversely, for LU the contribution of housing and mobility is higher in the top-down approach (respectively 25% and 5%) than in the bottom-up approach (20% for housing and below 5% for mobility); the elementary flows driving this difference are explored in the next section. Food is also the main contributing sector regarding PM in both approaches, yet with a different share of the total impacts (45% in the pLCA-CF and 30% in the IO-CF, still with an additional 7% due to Restaurants and Hotels in the IO-CF). Housing represents 30% in the pLCA-CF, compared to 28% in the IO-CF; in both cases, PM emissions are related mainly to the combustion of fuels used for space heating in European houses. Moreover, in both approaches mobility is the main driver for POF, yet with slightly different shares (40% in the pLCA-CF versus 32% in the IO-CF).

**Fig. 5:**
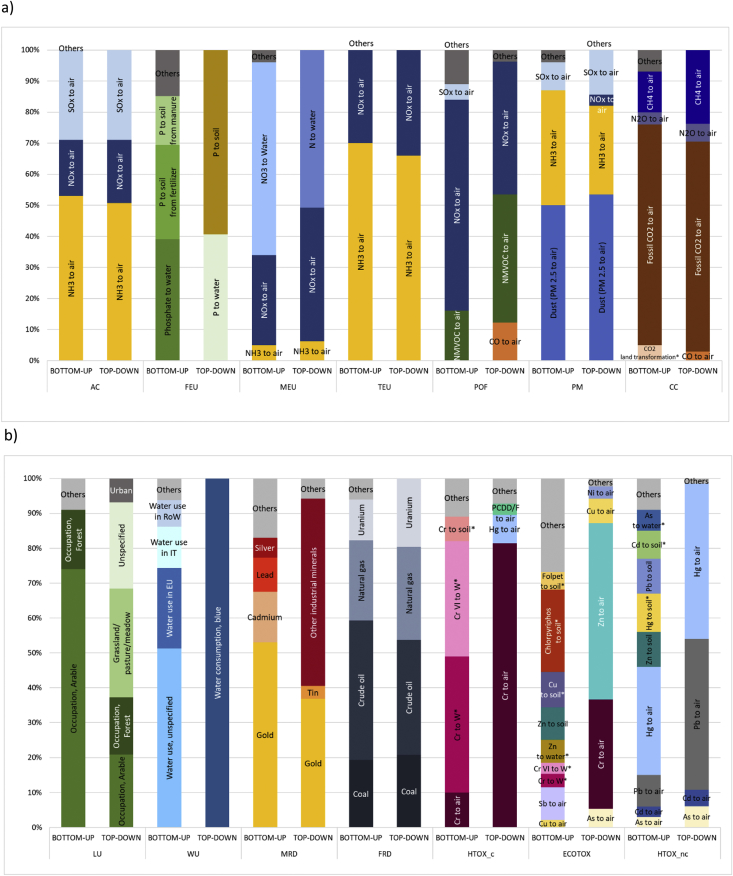
Contribution by substance (elementary flow) to the total pLCA-Consumer Footprint and IO-Consumer Footprint in Europe, presented in two groups: (a) AC; FEU, MEU, TEU, POF, PM and CC; (b) LU, WU, MRD, FRD, HTOX-c, ECOTOX and HTOX-nc. Note: Substances that are reported as contributors in the bottom-up approach but that are not reported in the environmental extensions of EXIOBASE 3 (and accordingly do not appear as contributors to the IO-CF because they are not accounted for) are marked with an asterisk (*) after the name. Elementary flows with contributions lower than 3% are aggregated in the category “Others”. The extended names of impact categories are reported in Table 1.

Finally, the identification of the most contributing sectors by the two approaches diverges significantly for the impact categories HTOX-nc, ECOTOX, and MRD. The impact on ECOTOX and HTOX-nc calculated with the bottom-up approach is dominated by the food sector, whereas the contribution to the impact calculated with the top-down approach shows a more distributed relevance of the COICOP divisions considered. Furthermore, the COICOP division Housing, water, electricity, gas and other fuels has a large contribution to the impact of the IO-CF on MRD. This is mostly due to expenditures on Real estate activities and Construction, which represent in total 24% of the impact of household consumption on MRD. On the contrary, the pLCA-CF allocates most of the impacts to Appliances and Mobility, because those are the sectors in which precious metals, responsible for the highest impact on MRD (Fig. 5), are used the most.

Overall, this identification of the areas of consumption most contributing to the impacts is in line with results of previous studies. Identifying food as a hotspot, even if it is not the main contributor to all the impact categories considered, is in line with what emerged from e.g. Ivanova et al. (2016). That study, adopting a top-down approach, found that food is the main contributor to land footprint, material footprint and water footprint of Europe, whereas mobility is the main contributor to carbon footprint. Similarly, Tukker et al. (2016) found that food products, motor vehicles, and buildings (including direct fuel emissions under households) have the highest footprints in EU-27. Kalbar et al. (2016) applied a bottom-up approach to calculate the impact of personal metabolism in Denmark and identified food as a hotspot for several impact categories, including land use, freshwater ecotoxicity, freshwater eutrophication and marine eutrophication (which have a correspondence with results found in the present study). Similarly, the identification of housing (including thermal energy use) as the most important contributor to fossil resource depletion is in line with results obtained in this study.

### 3.4 Hotspot analysis, by substances contributing to impact

The contribution analysis by substance, reported in Fig. 5, shows that there is a good to very good correspondence between the two approaches for some of the impact categories (namely AC, TEU, CC, PM and FRD). In particular, depending on the approach undertaken, NH_3_ emissions to air contribute to 51–53% of the impact on AC, and to 66–70% on TEU. Similarly, NOx emissions to air respectively contribute to 18–20% of the impact on AC, and to 30–34% on TEU. Very similar contributions are also observed regarding fossil CO_2_ impact contribution on CC (68%–71%), PM2.5 on PM (50%–53%) and coal, crude oil and natural gas impact contributions on FRD. These similar patterns in the contributions however come along with different values of impacts. In particular, regarding PM, it is noteworthy that the total amount of PM2.5 emissions to air generated by the Consumer Footprint is similar in both approaches (1.62*10^9^ kg in the IO-CF vs 1.77*10^9^ kg in the pLCA-CF). However, the impact generated according to the two approaches is different. This is mainly due to a difference in the specification of the sub-compartments where emissions occur in the two inventories, i.e. to the characterization factor associated to those emissions. In the pLCA-CF 58% of PM2.5 emissions occur in “low populated areas”, where the potential health effect is lower, and only 27% in “urban air, close to ground” (where the potential effect is higher, and this is accounted for with a characterization factor 21 times larger than if emitted to “low populated areas”). On the contrary, in the IO-CF no compartment of emission is distinguished for PM2.5, so emissions are totally accounted for as “unspecified”, and associated to the highest characterization factor available (that is, the characterization factor of emissions to “urban air, close to ground”). This difference in characterization factors, due to limited details in EXIOBASE 3 on PM2.5 emissions beyond the total amount, is the main driver for the factor two of difference observed between pLCA-CF and IO-CF regarding impact on PM (Table 1).

For the remaining impact categories, the contribution by substance is very different in the two approaches. Those differences are due to several reasons, primarily some substances are not included in the inventory used in the top-down approach (those substances are marked with an asterisk in Fig. 5), or implemented but with a lower level of details (e.g. regarding details on the emission compartment).

In the case of POF, FEU, MEU and MRD results of the two approaches show a similar list of substances most contributing to impacts, but to different extents. In the case of POF, the emissions of NMVOC and NOx to air are the main contributors in both approaches, but the relative importance of the two is different (regarding NMVOC, 41% in the top-down compared to 16% in the bottom-up). The larger amount of NMVOC emitted according to the top-down approach (7 times larger than in the pLCA-CF) explains 28% of the difference in the total impact calculated with the two approaches (Table 1).

Moreover, the main difference in the contribution of substances to FEU lies in the level of details in the nomenclature of flows, from one approach to the other. Emissions of P to soil contribute to 59% of the total in the IO-CF, compared to 46% in the pLCA-CF (in that case reported as P from fertilizer and P from manure). Similarly, around 40% of the pLCA-CF is due to the emission of phosphate to water. This contribution becomes 50% if the unspecified flow P to water is added to the previous one, quite comparable to the share due to the emission of P to water in the IO-CF (40%).

Regarding MEU, the largest share of the total impact is caused respectively by the emission of N to water (IO-CF, 51%) and by NO_3_ to water (pLCA-CF, 62%). This difference reflects the difference in the inventory values of the two approaches: 7.40*10^9^ kg N in IO-CF (51% of the total emissions of N) and 3.13*10^10^ kg NO_3,_ corresponding to 7.06*10^9^ kg N in pLCA-CF (62% of the total emissions of N). Finally, the total impact on MRD in the top-down approach is driven by the aggregated flow “Other industrial minerals” (54% of the total impact), by the use of gold (37%) and to a much lower extent of tin (4%). On the contrary, in the pLCA-CF, metals show the largest contributions (of most importance gold, 53%, and cadmium, 15%, but also lead and silver). However, at the inventory side the amount of gold accounted for in the pLCA-CF (2.61*10^5^ kg) is lower than it is in the IO-CF (1.75*10^6^ kg).

For the remaining impact categories (HTOX-c, HTOX-nc, ECOTOX, LU and WU), the two approaches do not converge in the identification of the most contributing substances, i.e. it is more difficult to identify substances to which priority should be assigned in the development of impact reduction measures. The analysis of the contribution by substance to toxicity-related impacts highlights that some of the substances that are of most relevance for the pLCA-CF are excluded from EXIOBASE 3, and accordingly from the IO-CF. In EXIOBASE 3 there are no emissions of pesticides, no emissions of metals to water and the only emission of metals to soil are emissions of zinc and lead. This difference mainly explains both the lower contribution of the food sector in the IO-CF (regarding HTOX-c, HTOX-nc and ECOTOX), and the lower impact of the IO-CF in absolute terms (regarding HTOX-c and ECOTOX; Table 1). In particular, focusing on HTOX_c and both approaches, the substance that contributes the most to the impact is chromium (Cr; see SI file S12 for similar discussions regarding HTOX-nc and ECOTOX). However, in the case of the IO-CF, 80% of the impact is generated by the emission of Cr to air, whereas in the pLCA-CF Cr to air contributes only for 10% to the total impact, while the emissions of Cr to water, both as Cr and Cr VI, and contribute to 40% and 30% respectively. The exclusion of Cr emissions to water from EXIOBASE 3 explains a major share of the lower absolute impact calculated with the top-down approach compared to the one calculated with the bottom-up (Table 1). In fact, when excluding the contribution of emissions of chromium to water, the impact calculated with the pLCA-CF is 1.98*10^4^ CTUh, which is closer to the one of the top-down without services (1.78*10^4^ CTUh), even if still slightly higher (11%).

Moreover, the impact on LU in the pLCA-CF is driven by occupation of arable land, which is far less relevant in the IO-CF. The difference in impact between the two approaches (with a factor four of difference, see Table 1) is primarily driven by two issues. Firstly, the model of the pLCA-CF does not include any occupation of grassland, pasture and meadows (whereas EXIOBASE 3, and subsequently the IO-CF, does) and, secondly, the total area occupied is four times lower in the inventory of the pLCA-CF (2.10*10^12^ m^2^, compared to 8.76*10^12^ m^2^ in the top-down). Finally, the top-down approach considers only one type of water use (blue water, as total), whereas in the bottom-up approach WU impacts are regionalized. However, most of the impact on WU in pLCA-CF is generated in macro-regions (Europe, Rest of the World – RoW and unspecified regions) for which a general characterization factor is applied, equal to the one used for the generic flow of blue water in IO-CF.

### 3.5 Relevance of use phase and direct emissions

**Fig. 6:**
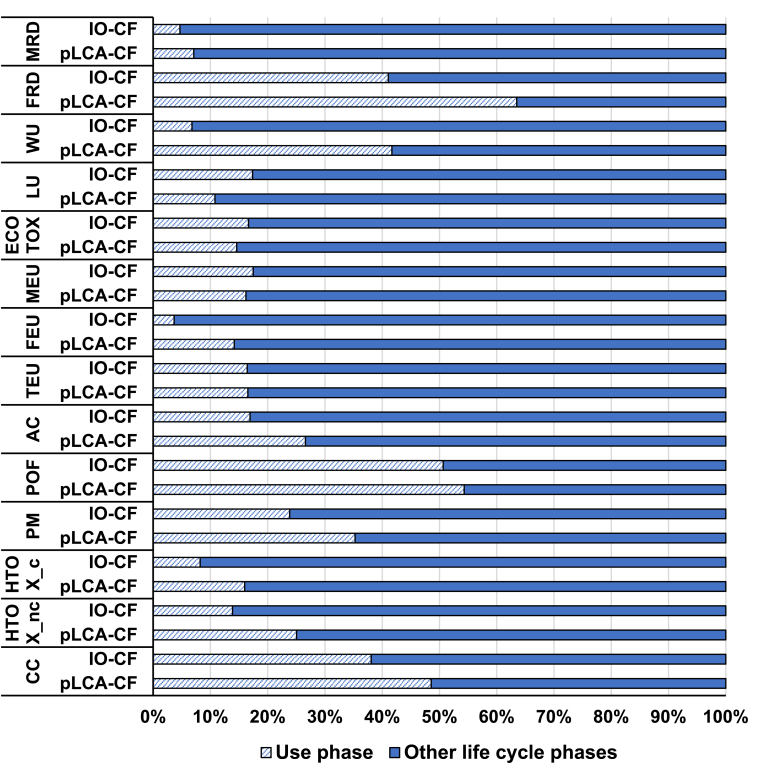
Contribution of the use phase over the total life-cycle impact calculated with the two approaches.

According to the results of the two approaches, for most of the impact categories the impact is driven by the supply chain of products consumed and used by European households (the comparison is shown in Fig. 6; absolute results are presented in Table S8). Those activities contribute to more than 80% of the impact on HTOX-c, TEU, FEU, MEU, ECOTOX, LU and MRD both in top-down and bottom-up approaches. Yet, regarding other impact categories, the contribution of the use phase is more significant. Firstly, the contribution of the use phase to HTOX-nc is 25% in the pLCA-CF and 14% in the IO-CF. For WU the contribution of the use phase (i.e. of water use in the houses of European citizens, for example due to the preparation of food or the use of detergents and soaps) is more than 40% of the total in the bottom-up approach, whereas it is below 10% in the top-down approach. Also for AC, the contribution of the use phase is higher in the bottom-up (26%) than in the top-down approach (17%). The contribution of the use phase to AC in the pLCA-CF is mostly generated by the emissions of nitrogen oxides from transport, and especially from air transport.

Other impacts to which the use phase (or direct emissions) is contributing significantly in both approaches are CC (about 50% in pLCA-CF and 40% in IO-CF), PM (35% and 24%), POF (53% and 51%), and FRD (63% and 40%). The main drivers of these contributions are the combustion of fuels (either for transport or housing heating) and the production of electricity used in the houses in both approaches. Even if the pLCA-CF and the IO-CF converge in indicating the use phase as relevant for FRD, they diverge in terms of the extent of its contribution to the total impact. The bottom-up approach shows a larger contribution of the use phase, mainly due to higher inventory values related to the use of fossil fuels for mobility and space heating in the buildings. Such difference in inventory values lies in the different modelling approaches, respectively based on statistics in the pLCA-CF (e.g. considering traffic intensity and composition of the fleet) and derived from the EXIOBASE data on the use of fossil fuels allocated between COICOP divisions in the IO-CF (see SI section S11 for more details).

### 3.6 Discussion on main convergences and divergences in results

The characterized impact of household consumption in Europe calculated with bottom-up and top-down approaches is quite different in absolute terms, with a difference that varies among impact categories. For PM, POF, LU and MRD, the impact of top-down is more than twice higher than the one of bottom-up (nine times higher in the case of PM and MRD). The exclusion of “services” in the bottom-up approach can explain only to some extent the difference of results from the two approaches. The use phase is contributing significantly to CC, PM, POF and FRD, with a different contribution in the two approaches especially in the case of FRD (63% versus 40%). In both cases, the main drivers of this contribution are the combustion of fuels (either for transport or heating of the houses) and the production of electricity used in the houses. The two approaches converge in identifying food consumption as a key driver of impacts regarding most impact categories, namely AC, TEU, FEU, MEU, PM, LU and WU (with a generally lower contribution in top-down compared to bottom-up). Housing and mobility are relevant as well in both approaches for some impact categories (in particular CC, POF, PM and FRD). Some substances are contributing to several impact categories, and are identified as hotspot by both approaches: emission of NH_3_ to air (for AC and TEU), of NOx to air (for AC, MEU, TEU and to some extent POF), of P to water and to soil (for FEU) and of fossil CO_2_ to air (for CC). Therefore, the use of both approaches to calculate the same indicator (Consumer Footprint) enabled identifying the main drivers of impact stemming from household consumption in EU. The key converging results from both approaches can be considered as a robust basis to support the definition of policies aimed at reducing the environmental footprint of household consumption in Europe.

Moreover, this study helped to quantify the differences in the calculation of the impact from household consumption in Europe, due to the differences in the two approaches. Results showed that the difference in scope (e.g. the exclusion of services in the pLCA-CF) only partly explains the differences in results, which instead are mainly driven by significant differences at the inventory side. Three main drivers of the differences have been observed: (*i*) differences in the intensity of emissions. For example, the level of emissions of NMVOC to air is much larger in the IO-CF, compared to the pLCA-CF (with a factor 7 of difference between the two approaches); (*ii*) differences in the coverage of elementary flows. For instance, the substances that are the main contributors to the impact on toxicity according to the pLCA-CF (pesticides and emissions of metals to water) are not included in the inventory of the IO-CF*;* (*iii*) differences in the level of details relative to elementary flows. For example, in the case of mineral resource depletion, the main difference is the level of detail between the two approaches, again at the inventory side: in the IO-CF the aggregated flow “other industrial minerals”, which is the main contributor to the impact, limits the possibility to interpret results and to identify the most relevant substances. Moreover, in the case of PM, the impact is twice larger in the IO-CF compared to the pLCA-CF, whereas the total amount of emissions of the main contributing substance (PM2.5) is similar in both approaches. The difference in impact is mainly a difference at the impact assessment step: in the absence of information on the location of PM2.5 emissions in EXIOBASE 3 (either in low-populated or urban areas, to ground or from a stack), a proxy characterization factor must be used to calculate their impact on PM. Those issues should be taken into consideration when interpreting results from both approaches in future studies, or when further combining the two approaches in a hybrid framework. These three main drivers of the differences between the pLCA-CF and IO-CF (intensity of emissions; coverage of elementary flows, and in particular of emissions contributing to toxicity; level of details relative to elementary flows, and in particular of aggregation relative to “other industrial minerals” in the IO approach) are aligned with what has been found in a similar exercise of comparison between the two calculation approaches applied to the EU trade (Beylot et al., 2019b).

Future assessment of the environmental footprint of European household consumption could be based on the hybridization of the two approaches, i.e. expanding process-based LCA by adding input-output data to cover the process cut-offs, as already proposed and analysed by previous studies but so far rarely applied at the level of the full consumption of a country (Nakamura and Nansai, 2016; Pomponi and Lenzen, 2018; Suh and Huppes, 2002; Strømman and Solli, 2008; Peters et al., 2010; Deng et al., 2011; Weinzettel et al., 2014).

## 4 Conclusions

This study gave interesting insights on the main differences and the robustness of results from the application of process-based LCA and environmentally extended input-output tables to model the impact of household consumption. The comparison is complemented by a discussion of the effect that the limits and specific features of each approach can have on final results.

The two approaches converge in identifying food consumption as a key driver of impacts (with a generally lower contribution in the IO-CF compared to pLCA-CF). Housing and mobility are relevant as well for some impact categories (e.g. particulate matter emissions and use of fossil resources). For PM, POF, LU and MRD, the impact of IO-CF is higher than the one of bottom-up. The exclusion of “services” in the pLCA-CF can explain only to some extent the difference of results from the two approaches. Results show that the use phase contributes significantly to CC, PM, POF and FRD, with a different contribution in the two approaches. In both cases, the main drivers of this contribution are the combustion of fuels (either for transport or heating of the houses) and the production of electricity used in the houses. Finally, some substances are contributing to several impact categories, and are identified as hotspot by both approaches: emission of NH_3_ to air (for AC and TEU), of NOx to air (for AC; MEU, TEU and to some extent POF), of P to water and to soil (for FEU) and of fossil CO_2_ to air (for CC).

Main limitations of both approaches applied in this study were already known. However, in this study we report them more systematically. The limitations refer mainly on one hand to the more limited coverage of activities of the bottom-up approach (which does not account for the whole economy, but only for some sectors, in particular excluding services) and on the other hand to both the lower granularity of the top-down approach, which limits the possibility to analyse the role of products more in detail, and to the limited coverage of elementary flows. This calls for extending ongoing efforts on hybridisation of top-down approaches for macro-scale applications and to complement list of products in the bottom up approach to better capture and represent missing areas of consumption.
