# Supplementary material for: Environmental impacts of household consumption in Europe: Comparing process-based LCA and environmentally extended input-output analysis
Source: J Clean Prod. 2019 Dec 10;240:117966. doi: 10.1016/j.jclepro.2019.117966 (PMC6886559; doi:10.1016/j.jclepro.2019.117966)
Supplement: Multimedia component 1 [file mmc1.docx]

**SUPPORTING INFORMATION**

**Environmental impacts of household consumption in Europe: comparing process-based LCA and environmentally extended input-output analysis**

Valentina Castellani^a^, Antoine Beylot^a^, Serenella Sala^a^*

^a^European Commission-Joint Research Centre, Via Enrico Fermi 2749, I-21027 Ispra (VA), Italy

Contents

[**Environmental impacts of household consumption in Europe: comparing process-based LCA and environmentally extended input-output analysis** 1](#_Toc14800406)

[S 1. Life Cycle phases included in the BoPs 2](#_Toc14800407)

[S 2. Building archetypes in BoP Housing 3](#_Toc14800408)

[S 3. Means of transport in BoP Mobility 4](#_Toc14800409)

[S 4. Representative products in BoP Food 5](#_Toc14800410)

[S 5. Representative products in BoP Appliances 6](#_Toc14800411)

[S 6. Representative products in BoP Household goods 7](#_Toc14800412)

[S 7. Contribution by areas of consumption 9](#_Toc14800413)

[S 8. Contribution of the use phase 11](#_Toc14800414)

[S 9. Allocation to COICOP divisions 12](#_Toc14800415)

[S 10. Classification of services 13](#_Toc14800416)

[S 11. Calculation of the impacts associated with the use phase in the IO-CF 18](#_Toc14800417)

[S 12. Hotspot analysis, by substance: focus on toxicity and ecotoxicity-related impacts 19](#_Toc14800418)

S 1. Life Cycle phases included in the BoPs

*Table S1. Life Cycle phases and activities included in the five BoPs composing the pLCA-Consumer Footprint*

| **Life Cycle phase** | **BoP Housing** | **BoP Mobility** | **BoP Food** | **BoP Household goods** | **BoP Appliances** |
| --- | --- | --- | --- | --- | --- |
| Upstream | Production of construction materials | Construction of mobility infrastructure (roads, railway, airports) | Agricultural activities: cultivation of crops and animal rearing | Manufacture of product components | Manufacture of product components |
| Packaging | *(not applicable)* | *(not applicable)* | Production of packaging materials; EoL of packaging materials | Production of packaging materials;  EoL of packaging materials | Production of packaging materials;  EoL of packaging materials |
| Logistics | *(not applicable)* | *(not applicable)* | Storage at distribution centre (whenever relevant); transport from production site to retail; storage at retail (for refrigerated products) | Transport to retail | Transport to retail |
| Use | Energy and water use by dwelling’s users; wastewater treatment | Production of fuel; emissions related to vehicle use (e.g. from combustion of fuel in internal combustion engines); tire wear emissions, brake wear emissions; road wear emissions. | Transport of product from retail to user; cooking (whenever relevant), refrigerated storage of products at home | Transport of product from retail to user; electricity use (whenever relevant); water use (e.g. for detergents); | Transport of product from retail to user; electricity use; water use (e.g. for dishwashers and washing machines), wastewater treatment |
| Maintenance | Production of components substituted during the building lifetime (e.g. windows); EoL of substituted components | Production of components substituted during the vehicle lifetime (e.g. tires); EoL of substituted components | *(not applicable)* | *(not applicable)* | Production of components substituted during the appliance lifetime; EoL of substituted components |
| End of life | Building demolition; sorting of materials; treatment of sorted materials (including recycling) | Vehicle demolition; sorting of materials; treatment of sorted materials (including recycling) | Final disposal of food waste; wastewater treatment and auxiliary processes due to toilet use | Wastewater treatment (for detergents, personal care products and toilet paper) | Wastewater treatment (for washing machines and dishwashers) |

S 2. Building archetypes in BoP Housing

The basket is composed by 24 reference dwellings, representative of the EU-27 housing stock in the year 2010, divided by type of building, climate zone and year of construction. The features chosen to define the representative dwellings in the BoP-housing are:

- the dwelling type: Multi-family house (MFH) or Single- Family House (SFH)
- the climate of the area in which the building is located: cold, moderate or warm
- the period of construction: before 1945, between 1946 and 1969, between 1970 and 1989, between 1990 and 2010.

*Table S2. Number of dwellings by dwelling archetype and number of dwellers by climate area in the baseline model of the BoP Housing*

| **Building type** | **Climate area** | **Year of construction** | **Total number of dwellings** | **Total number of dwellers** |
| --- | --- | --- | --- | --- |
| **SFH** | WARM | <1945 | 3,990,078 | 54,801,521 |
|  |  | 1945-1969 | 3,940,268 |  |
|  |  | 1970-1989 | 5,029,842 |  |
|  |  | 1990-2008 | 3,015,954 |  |
|  | MODERATE | <1945 | 19,053,376 | 221,050,717 |
|  |  | 1945-1969 | 21,741,474 |  |
|  |  | 1970-1989 | 24,874,549 |  |
|  |  | 1990-2008 | 15,835,402 |  |
|  | COLD | <1945 | 1,137,005 | 11,733,022 |
|  |  | 1945-1969 | 1,123,212 |  |
|  |  | 1970-1989 | 1,258,137 |  |
|  |  | 1990-2008 | 629,666 |  |
| **MFH** | WARM | <1945 | 5,563,385 | 72,640,110 |
|  |  | 1945-1969 | 10,977,814 |  |
|  |  | 1970-1989 | 12,326,198 |  |
|  |  | 1990-2008 | 6,923,950 |  |
|  | MODERATE | <1945 | 12,883,862 | 125,289,263 |
|  |  | 1945-1969 | 16,543,072 |  |
|  |  | 1970-1989 | 19,849,947 |  |
|  |  | 1990-2008 | 11,961,082 |  |
|  | COLD | <1945 | 1,326,949 | 9,463,779 |
|  |  | 1945-1969 | 1,580,981 |  |
|  |  | 1970-1989 | 1,831,828 |  |
|  |  | 1990-2008 | 910,548 |  |

S 3. Means of transport in BoP Mobility

A selection of 27 representative means of transport was made to represent the fleet composition in Europe in 2010 in the BoP Mobility, as follows:

- 9 types of Gasoline passenger cars (with 3 engine capacities: <1.4L, between 1.4-2.0L and >2.0L, and 3 emissions standards: Euro 0-Euro 3, Euro 4 and Euro 5)
- 6 types of Diesel passenger cars (with 2 engine capacities: between 1.4-2.0L and >2.0L, and 3 emissions standards: Euro 0-Euro 3, Euro 4 and Euro 5)
- LPG passenger car
- 3 types of 2-wheelers (mopeds, motorcycles <250cc, motorcycles >250cc)
- 3 types of buses (diesel urban buses, CNG urban buses and coaches)
- 2 types of trains (electric and diesel)
- 3 types of flights (national, intra-EU and extra-EU)

*Table S3. Representative means of transport and km travelled per year in Europe, as in the baseline model of the BoP Mobility*

| **Means of transport** | | **Representative product** | | **Vehicle- km (million)** | **Passenger-km (million)** |
| --- | --- | --- | --- | --- | --- |
| Road transport | Passenger Cars | Gasoline <1.4 L | Euro 0, 1, 2, 3 | 588,267 | - |
|  |  | Gasoline <1.4 L | Euro 4 | 112,794 | - |
|  |  | Gasoline <1.4 L | Euro 5 | 74,617 | - |
|  |  | Gasoline 1.4 - 2.0 L | Euro 0, 1, 2, 3 | 530,852 | - |
|  |  | Gasoline 1.4 - 2.0 L | Euro 4 | 101,344 | - |
|  |  | Gasoline 1.4 - 2.0 L | Euro 5 | 67,043 | - |
|  |  | Gasoline >2.0 L | Euro 0, 1, 2, 3 | 97,936 | - |
|  |  | Gasoline >2.0 L | Euro 4 | 18,762 | - |
|  |  | Gasoline >2.0 L | Euro 5 | 12,412 | - |
|  |  | Diesel 1.4 - 2.0 L | Euro 0, 1, 2, 3 | 816,541 | - |
|  |  | Diesel 1.4 - 2.0 L | Euro 4 | 155,884 | - |
|  |  | Diesel 1,4 - 2.0 L | Euro 5 | 103,123 | - |
|  |  | Diesel >2.0 L | Euro 0, 1, 2, 3 | 207,188 | - |
|  |  | Diesel >2.0 L | Euro 4 | 39,554 | - |
|  |  | Diesel >2.0 L | Euro 5 | 26,166 | - |
|  |  | LPG | Euro 0, 1, 2, 3, 4, 5 | 48,971 | - |
|  | 2W | Mopeds <50 cc | Euro 0, 1, 2, 3 | 48,168 | - |
|  |  | Motorcycles <250 cc | Euro 0, 1, 2, 3 | 22,440 | - |
|  |  | Motorcycles >250 cc | Euro 0, 1, 2, 3 | 44,377 | - |
|  | Buses | Urban Buses 15 - 18 t | Euro 0, 1, 2, 3 | 24,971 | - |
|  |  | Coaches <=18 t | Euro 0, 1, 2, 3 | 2,288 | - |
|  |  | Urban CNG Buses | Euro 1, 2, 3 | 2,288 | - |
| Rail transport | | Electric | | - | 286,014 |
|  |  | Diesel | | - | 114,581 |
| Air transport | | National flights | | - | 121,434 |
|  |  | Intra-EU flights | | - | 726,695 |
|  |  | Extra-EU flights | | - | 1862,897 |
| **Total** | |  | | **3,145,985** | **2,180,173** |

S 4. Representative products in BoP Food

The BoP Food consists of a basket of the most relevant food product groups, selected by importance in mass and economic value, to depict the average consumption for nutrition of EU citizens in 2010. The BoP Food also includes products that are representative of emerging food consumption trends and types of food and beverages whose consumption has been increasing during the past decade (e.g. tofu, pre-prepared meals, etc.).

*Table S4. Representative products with related baseline and upscaled quantities (per person) in the baseline model of the BoP Food*

| **Product Group** | **Representative products** | **Per-capita consumption (kg/pers.*yr^-1^)** | **Share of EU consumption covered by representative products (%)** | **Per-capita consumption upscaled (kg/pers.*yr^-1^)** |
| --- | --- | --- | --- | --- |
| MEAT | Pork meat | 41.0 | 88.8% | 46.2 |
|  | Beef meat | 13.7 |  | 15.4 |
|  | Poultry meat | 22.0 |  | 24.8 |
| SEAFOOD | Farmed salmon | 1.9 | 85.9% | 2.2 |
|  | Cod (wild) | 5.6 |  | 6.5 |
|  | Shrimps | 1.4 |  | 1.6 |
| DAIRY | Milk | 79.7 | 75.3% | 106 |
|  | Cheese | 15 |  | 19.9 |
|  | Butter | 3.6 |  | 4.8 |
| EGGS | Eggs | 13.2 | 100% | 13.2 |
| CEREAL-BASED PRODUCTS | Bread | 39.3 | 72.1% | 54.5 |
|  | Pasta | 8.2 |  | 11.4 |
|  | Rice | 9 |  | 12.5 |
| SUGAR | Sugar | 27.8 | 62.9% | 44.2 |
| OILS | Sunflower oil | 5.3 | 24.4% | 21.8 |
|  | Olive oil | 5.3 |  | 21.7 |
| TUBERS | Potatoes | 69.1 | 50.6% | 137.0 |
| VEGETABLES | Tomatoes | 12.8 | 50.6% | 25.3 |
| LEGUMES | Beans | 2.3 | 34.9% | 6.6 |
|  | Tofu | 4.3 | 100% | 4.3 |
| FRUITS | Oranges | 17.4 | 22.9% | 76.0 |
|  | Apples | 16.1 |  | 70.3 |
|  | Bananas | 10.3 |  | 45.0 |
| NUTS | Almonds | 0.6 | 35.8% | 1.7 |
| COFFEE & TEA | Coffee | 3.5 | 42.9% | 5.9 |
|  | Tea | 0.8 | 80.1% | 1.0 |
| BEVERAGES | Beer | 69.8 | 92.7% | 75.3 |
|  | Wine | 24 |  | 25.9 |
|  | Mineral water | 105 | 42.9% | 245 |
| CONFECTIONERY PRODUCTS | Biscuits | 6.9 | 72.1% | 9.6 |
|  | Chocolate | 4.1 | 24.8% | 16.5 |
| PRE-PREPARED MEALS | Meat based dishes | 2.9 | 100% | 2.9 |
| **Total** | | **642** | **-** | **1,155** |

S 5. Representative products in BoP Appliances

The BoP Appliances consist of a process-based LCI model for a basket of products that represent the most relevant household appliances in terms of energy consumption and market share in Europe. The amount of representative products included in the BoP Appliances is calculated starting from the analyses of the existing stock done for the Ecodesign preparatory studies. For each representative product, the entire stock present in European households is allocated to the reference year (dividing it by the number of service life years of the representative product chosen), and then to the number of users (i.e. European citizens in the reference year). This results in a per-capita yearly consumption expressed in pieces/person*year^-1^.

*Table S5. Representative products with related baseline and upscaled quantities (per person) in the baseline model of the BoP Appliances*

| **Product Group** | **Representative Products** | **Per-capita yearly consumption (pieces/pers.*yr^-1^)** | **Share of the EU stock covered by representative products (%)** | **Per-capita yearly consumption upscaled (pieces/pers.*yr^-1^)** |
| --- | --- | --- | --- | --- |
| Dishwashing machine | Dishwasher 10 ps | 0.00198 | 100% | 0.00198 |
|  | Dishwasher 13 ps | 0.0112 |  | 0.0112 |
| Washing and drying machine | Washing Machine | 0.0296 | 100% | 0.0296 |
|  | Electric condenser tumble dryer | 0.00579 | 60% | 0.00965 |
| Refrigeration | Combined refrigerators-freezers | 0.0222 | 56% | 0.0397 |
| Air conditioning | Air conditioner, single split | 0.00373 | 100% | 0.00373 |
| Domestic cooking appliances | Electric oven (built-in) | 0.0103 | 46% | 0.0223 |
| Lighting | Compact fluorescent lamp | 0.246 | 100% | 0.246 |
|  | Halogen lamp, low voltage | 0.408 |  | 0.408 |
|  | Halogen lamp, mains voltage | 0.638 |  | 0.638 |
|  | Incandescent lamp | 0.648 |  | 0.648 |
| Computer | Notebook | 0.0284 | 40% | 0.0711 |
| TV screen | LCD TV screen | 0.0584 | 53% | 0.11 |

S 6. Representative products in BoP Household goods

The BoP Household goods consists of a process-based LCI model for a basket of products that represent the most relevant product groups consumed in households. The selection of the product groups to be included in the basket was based mainly on the list of product groups already covered by the Ecolabel and for which Green Public Procurement (GPP) criteria were available, complemented with the product groups for which a Product Environmental Footprint (PEF) pilot was ongoing. The reason of this choice is that the selection of product groups that are covered by Ecolabel or GPP criteria follows a set of criteria (including market significance in terms of stock volume and sales and importance of the environmental impact generated) that is in line with the ones that drove the selection of the representative products for the other BoPs.

*Table S6. Representative products with related baseline and upscaled quantities (in kg, pieces or pair, depending on the product group) in the baseline model of the BoP Household goods*

| **Product Group** | **Representative product** | **Per-capita apparent consumption**  **(unit/pers*year^-1^)** | **Coverage by representative products** | **Per capita apparent consumption upscaled (unit/pers*year^-1^)** | **Unit** |
| --- | --- | --- | --- | --- | --- |
| Detergents | All-Purpose Cleaners and Sanitary Cleaners | 9.99 | 100% | 9.99 | kg |
|  | Detergents for Dishwashers | 2.43 |  | 2.43 | kg |
|  | Hand Dishwashing Detergents | 1.75 |  | 1.75 | kg |
|  | Laundry Detergents liquid | 10.03 |  | 10.03 | kg |
|  | Laundry Detergents powder | 3.10 |  | 3.10 | kg |
| Absorbent Hygiene products (sanitary products) | Baby diapers | 1.57 | 49% | 3.22 | kg |
|  | Sanitary pads | 1.97 |  | 4.05 | kg |
|  | Tampons | 0.04 |  | 0.09 | kg |
|  | Breast pads | 0.27 |  | 0.56 | kg |
| Rinse-off cosmetics | Bar soap | 1.43 | 31% | 4.59 | kg |
|  | Liquid soap | 0.57 |  | 1.82 | kg |
|  | Shampoo | 0.98 |  | 3.13 | kg |
|  | Hair conditioner | 0.65 |  | 2.07 | kg |
| Furniture | Bedroom furniture | 0.131 | 66% | 0.20 | p |
|  | Kitchen furniture | 0.198 |  | 0.30 | p |
|  | Upholstered seat | 0.124 |  | 0.19 | p |
|  | Non-Upholstered seat (wooden seat) | 0.179 |  | 0.27 | p |
|  | Dining room furniture | 0.109 |  | 0.17 | p |
| Bed mattresses | Mattress (Latex, PUR and spring mattresses) | 0.079 | 89% | 0.09 | p |
| Footwear | Work and Waterproof (WW) | 0.24 | 50% | 0.48 | pa |
|  | Sport | 0.30 |  | 0.61 | pa |
|  | Leisure | 1.15 |  | 2.29 | pa |
|  | Fashion | 1.15 |  | 2.29 | pa |
| Textile products (clothes) | T-shirt | 7.03 | 22% | 31.80 | p |
|  | Women blouse | 1.89 |  | 8.55 | p |
|  | Men trousers | 0.83 |  | 3.74 | p |
|  | Jeans | 1.06 |  | 4.79 | p |
| Paper products | Newspaper | 45.99 | 31% | 90.50 | kg |
|  | Book | 5.91 |  | 19.26 | kg |
|  | Toilet paper | 7.23 |  | 23.55 | kg |

S 7. Contribution by areas of consumption

*Table S7. Contribution by areas of consumption (represented by BoPs in the pLCA-CF and COICOP divisions in the IO-CF)*

|  | | | Food | | | | Household goods and appliances | | | |
| --- | --- | --- | --- | --- | --- | --- | --- | --- | --- | --- |
| Impact category | Unit | Indicator | Food and non-alcoholic beverages | Alcoholic beverages, tobacco and narcotics | Restaurants and hotels | Tot Food | Clothing and footwear | Miscellaneous goods and services | Furnishings, household equipment and routine household maintenance | Tot Household goods and appliances |
| **Climate change** | kg CO_2_ eq | pLCA-CF | - | - | - | 1.59E+12 | - | - | - | 6.52E+11 |
|  |  | IO-CF | 1.13E+12 | 2.46E+10 | 3.04E+11 | 1.46E+12 | 1.70E+11 | 1.54E+11 | 2.10E+11 | 5.33E+11 |
| **Ozone depletion** | kg CFC11 eq | pLCA-CF | - | - | - | 2.68E+06 | - | - | - | 8.91E+04 |
|  |  | IO-CF | - | - | - |  | - | - | - | - |
| **Human toxicity, non-cancer** | CTUh | pLCA-CF | - | - | - | 1.10E+05 | - | - | - | 3.91E+04 |
|  |  | IO-CF | 5.84E+04 | 2.65E+03 | 2.03E+04 | 8.13E+04 | 1.74E+04 | 1.58E+04 | 4.92E+04 | 8.23E+04 |
| **Human toxicity, cancer** | CTUh | pLCA-CF | - | - | - | 1.96E+04 | - | - | - | 2.14E+04 |
|  |  | IO-CF | 2.80E+03 | 1.28E+02 | 8.81E+02 | 3.81E+03 | 7.69E+02 | 7.39E+02 | 2.53E+03 | 4.04E+03 |
| **Particulate matter** | Disease incidence | pLCA-CF | - | - | - | 1.61E+05 | - | - | - | 3.85E+04 |
|  |  | IO-CF | 2.16E+05 | 2.80E+03 | 5.05E+04 | 2.70E+05 | 2.61E+04 | 1.91E+04 | 3.49E+04 | 8.01E+04 |
| **Ionising radiation** | kBq U^235^ eq | pLCA-CF | - | - | - | 3.07E+10 | - | - | - | 3.05E+10 |
|  |  | IO-CF | - | - | - | - | - | - | - | - |
| **Photochemical ozone formation** | kg NMVOC eq | pLCA-CF | - | - | - | 2.72E+09 | - | - | - | 2.03E+09 |
|  |  | IO-CF | 6.79E+09 | 1.11E+08 | 1.42E+09 | 8.32E+09 | 1.37E+09 | 2.05E+09 | 1.55E+09 | 4.98E+09 |
| **Acidification** | molc H^+^ eq | pLCA-CF | - | - | - | 2.20E+10 | - | - | - | 3.67E+09 |
|  |  | IO-CF | 2.68E+10 | 1.76E+08 | 5.22E+09 | 3.22E+10 | 1.66E+09 | 1.32E+09 | 1.71E+09 | 4.69E+09 |
| **Eutrophication, terrestrial** | molc N eq | pLCA-CF | - |  |  | 9.43E+10 | - | - | - | 7.78E+09 |
|  |  | IO-CF | 1.15E+11 | 3.91E+08 | 2.09E+10 | 1.36E+11 | 4.75E+09 | 3.51E+09 | 2.27E+09 | 1.05E+10 |
| **Eutrophication, freshwater** | kg P eq | pLCA-CF | - | - | - | 2.95E+08 | - | - | - | 6.92E+07 |
|  |  | IO-CF | 2.78E+08 | 3.69E+05 | 5.44E+07 | 3.33E+08 | 9.39E+06 | 3.78E+06 | 2.00E+06 | 1.52E+07 |
| **Eutrophication, marine** | kg N eq | pLCA-CF | - | - | - | 8.75E+09 | - | - | - | 8.45E+08 |
|  |  | IO-CF | 7.99E+09 | 4.30E+07 | 1.47E+09 | 9.51E+09 | 1.97E+08 | 2.16E+08 | 1.92E+08 | 6.05E+08 |
| **Ecotoxicity, freshwater** | CTUe | pLCA-CF | - | - | - | 5.03E+12 | - | - | - | 8.91E+11 |
|  |  | IO-CF | 6.56E+10 | 2.99E+09 | 2.00E+10 | 8.86E+10 | 1.74E+10 | 1.72E+10 | 5.69E+10 | 9.14E+10 |
| **Land use** | Pt | pLCA-CF | - | - | - | 1.82E+14 | - | - | - | 2.81E+13 |
|  |  | IO-CF | 3.68E+14 | 3.58E+12 | 1.00E+14 | 4.72E+14 | 6.10E+13 | 1.75E+13 | 5.85E+13 | 1.37E+14 |
| **Water use** | m^3^ water eq | pLCA-CF | - | - | - | 3.11E+12 | - | - | - | 7.97E+11 |
|  |  | IO-CF | 2.41E+12 | 4.70E+10 | 7.12E+11 | 3.17E+12 | 9.86E+10 | 6.88E+10 | 7.80E+10 | 2.45E+11 |
| **Resource use, fossils** | MJ | pLCA-CF | - | - | - | 8.95E+12 | - | - | - | 8.80E+12 |
|  |  | IO-CF | 7.67E+12 | 3.85E+11 | 2.28E+12 | 1.03E+13 | 1.82E+12 | 2.46E+12 | 2.73E+12 | 7.02E+12 |
| **Resource use, mineral and metals** | kg Sb eq | pLCA-CF | - | - | - | 1.69E+06 | - | - | - | 1.25E+07 |
|  |  | IO-CF | 3.08E+07 | 1.26E+06 | 1.16E+07 | 4.37E+07 | 1.01E+07 | 2.59E+07 | 2.00E+07 | 5.59E+07 |

*Table S7. Contribution by areas of consumption (represented by BoPs in the pLCA-CF and COICOP divisions in the IO-CF) (continued)*

|  | | | Housing | Mobility |  |  |  |  |  |
| --- | --- | --- | --- | --- | --- | --- | --- | --- | --- |
| Impact category | Unit | Indicator | Housing, water, electricity, gas and other fuels | Transport | Health | Communications | Recreation and culture | Education | Total Footprint |
| **Climate change** | kg CO_2_ eq | pLCA-CF | 1.31E+12 | 1.25E+12 | - | - | - | - | 4.80E+12 |
|  |  | IO-CF | 1.71E+12 | 1.40E+12 | 5.22E+10 | 7.98E+10 | 2.28E+11 | 1.77E+10 | 5.48E+12 |
| **Ozone depletion** | kg CFC11 eq | pLCA-CF | 1.58E+05 | 2.91E+05 | - | - | - | - | 3.22E+06 |
|  |  | IO-CF | - | - | - | - | - | - |  |
| **Human toxicity, non-cancer** | CTUh | pLCA-CF | 5.75E+04 | 4.52E+04 | - | - | - | - | 2.51E+05 |
|  |  | IO-CF | 1.27E+05 | 1.20E+05 | 7.10E+03 | 8.80E+03 | 3.87E+04 | 2.13E+03 | 4.68E+05 |
| **Human toxicity, cancer** | CTUh | pLCA-CF | 1.70E+04 | 1.33E+04 | - | - | - | - | 7.14E+04 |
|  |  | IO-CF | 5.28E+03 | 5.78E+03 | 3.42E+02 | 4.04E+02 | 1.91E+03 | 1.01E+02 | 2.17E+04 |
| **Particulate matter** | Disease incidence | pLCA-CF | 1.18E+05 | 4.97E+04 | - | - | - | - | 3.68E+05 |
|  |  | IO-CF | 2.01E+05 | 1.21E+05 | 7.13E+03 | 9.37E+03 | 3.26E+04 | 2.23E+03 | 7.23E+05 |
| **Ionising radiation** | kBq U^235^ eq | pLCA-CF | 9.85E+10 | 8.38E+10 | - | - | - | - | 2.43E+11 |
|  |  | IO-CF | - | - | - | - | - | - | - |
| **Photochemical ozone formation** | kg NMVOC eq | pLCA-CF | 3.17E+09 | 5.12E+09 | - | - | - | - | 1.30E+10 |
|  |  | IO-CF | 1.03E+10 | 1.20E+10 | 3.49E+08 | 4.36E+08 | 1.07E+09 | 7.63E+07 | 3.76E+10 |
| **Acidification** | molc H^+^ eq | pLCA-CF | 6.47E+09 | 5.09E+09 | - | - | - | - | 3.73E+10 |
|  |  | IO-CF | 9.36E+09 | 9.48E+09 | 4.65E+08 | 5.44E+08 | 1.74E+09 | 1.45E+08 | 5.87E+10 |
| **Eutrophication, terrestrial** | molc N eq | pLCA-CF | 8.93E+09 | 1.51E+10 | - | - | - | - | 1.26E+11 |
|  |  | IO-CF | 1.94E+10 | 3.02E+10 | 9.22E+08 | 1.16E+09 | 2.87E+09 | 3.84E+08 | 2.01E+11 |
| **Eutrophication, freshwater** | kg P eq | pLCA-CF | 6.59E+07 | 3.65E+07 | - | - | - | - | 4.66E+08 |
|  |  | IO-CF | 1.78E+07 | 5.30E+06 | 1.49E+06 | 7.80E+05 | 2.70E+06 | 6.73E+05 | 3.77E+08 |
| **Eutrophication, marine** | kg N eq | pLCA-CF | 8.12E+08 | 1.38E+09 | - | - | - | - | 1.18E+10 |
|  |  | IO-CF | 1.50E+09 | 2.51E+09 | 7.27E+07 | 9.98E+07 | 2.35E+08 | 3.01E+07 | 1.46E+10 |
| **Ecotoxicity, freshwater** | CTUe | pLCA-CF | 5.54E+11 | 9.89E+11 | - | - | - | - | 7.46E+12 |
|  |  | IO-CF | 1.52E+11 | 1.42E+11 | 7.71E+09 | 9.77E+09 | 4.30E+10 | 2.29E+09 | 5.37E+11 |
| **Land use** | Pt | pLCA-CF | 2.77E+13 | 9.86E+12 | - | - | - | - | 2.48E+14 |
|  |  | IO-CF | 2.45E+14 | 3.64E+13 | 5.89E+12 | 5.34E+12 | 3.61E+13 | 2.27E+12 | 9.40E+14 |
| **Water use** | m^3^ water eq | pLCA-CF | 2.92E+12 | 2.19E+11 | - | - | - | - | 7.05E+12 |
|  |  | IO-CF | 4.22E+11 | 1.81E+11 | 2.65E+10 | 2.47E+10 | 9.78E+10 | 9.13E+09 | 4.18E+12 |
| **Resource use, fossils** | MJ | pLCA-CF | 2.33E+13 | 1.90E+13 | - | - | - | - | 6.00E+13 |
|  |  | IO-CF | 2.20E+13 | 1.91E+13 | 8.15E+11 | 1.20E+12 | 3.32E+12 | 2.87E+11 | 6.41E+13 |
| **Resource use, mineral and metals** | kg Sb eq | pLCA-CF | 2.50E+06 | 8.56E+06 | - | - | - | - | 2.52E+07 |
|  |  | IO-CF | 7.97E+07 | 3.39E+07 | 5.44E+06 | 6.82E+06 | 1.88E+07 | 1.88E+06 | 2.46E+08 |

S 8. Contribution of the use phase

*Table S8. Contribution of the use phase and of other life cycle phases to the pLCA-Consumer Footprint and the IO-Consumer Footprint*

| **Impact category** | | | **Use phase** | **Other life cycle phases** |
| --- | --- | --- | --- | --- |
| **Climate change** | **CC** | pLCA-CF | 4.64E+03 | 4.91E+03 |
|  |  | IO-CF | 2.09E+12 | 3.39E+12 |
| **Human toxicity, non-cancer** | **HTOX_nc** | pLCA-CF | 1.25E-04 | 3.75E-04 |
|  |  | IO-CF | 9.91E+04 | 6.16E+05 |
| **Human toxicity, cancer** | **HTOX_c** | pLCA-CF | 2.27E-05 | 1.19E-04 |
|  |  | IO-CF | 1.78E+03 | 1.99E+04 |
| **Particulate matter** | **PM** | pLCA-CF | 2.58E-04 | 4.74E-04 |
|  |  | IO-CF | 1.72E+05 | 5.51E+05 |
| **Photochemical ozone formation** | **POF** | pLCA-CF | 1.41E+01 | 1.19E+01 |
|  |  | IO-CF | 1.90E+10 | 1.86E+10 |
| **Acidification** | **AC** | pLCA-CF | 1.97E+01 | 5.44E+01 |
|  |  | IO-CF | 9.93E+09 | 4.87E+10 |
| **Eutrophication, terrestrial** | **TEU** | pLCA-CF | 4.15E+01 | 2.09E+02 |
|  |  | IO-CF | 3.31E+10 | 1.68E+11 |
| **Eutrophication, freshwater** | **FEU** | pLCA-CF | 1.31E-01 | 7.96E-01 |
|  |  | IO-CF | 1.38E+07 | 3.63E+08 |
| **Eutrophication, marine** | **MEU** | pLCA-CF | 3.80E+00 | 1.96E+01 |
|  |  | IO-CF | 2.54E+09 | 1.20E+10 |
| **Ecotoxicity, freshwater** | **ECOTOX** | pLCA-CF | 2.17E+03 | 1.27E+04 |
|  |  | IO-CF | 8.94E+10 | 4.47E+11 |
| **Land use** | **LU** | pLCA-CF | 5.33E+04 | 4.41E+05 |
|  |  | IO-CF | 1.63E+14 | 7.77E+14 |
| **Water use** | **WU** | pLCA-CF | 5.85E+03 | 8.18E+03 |
|  |  | IO-CF | 2.83E+11 | 3.89E+12 |
| **Resource use, fossils** | **FRD** | pLCA-CF | 7.57E+04 | 4.36E+04 |
|  |  | IO-CF | 2.63E+13 | 3.77E+13 |
| **Resource use, mineral and metals** | **MRD** | pLCA-CF | 3.55E-03 | 4.66E-02 |
|  |  | IO-CF | 1.15E+07 | 2.35E+08 |

S 9. Allocation to COICOP divisions

Tables reporting the allocation rules adopted for this study, excel S9 as separated file

S 10. Classification of services

Tables illustrating the classification of services adopted in this study

| **List of services quantified in Meuros in EXIOBASE 3 (Category "SERVICES")** | Unit |
| --- | --- |
| Retail trade services of motor fuel | Meuro |
| Wholesale trade and commission trade services; except of motor vehicles and motorcycles (51) | Meuro |
| Retail trade services; except of motor vehicles and motorcycles; repair services of personal and household goods (52) | Meuro |
| Supporting and auxiliary transport services; travel agency services (63) | Meuro |
| Post and telecommunication services (64) | Meuro |
| Financial intermediation services; except insurance and pension funding services (65) | Meuro |
| Insurance and pension funding services; except compulsory social security services (66) | Meuro |
| Services auxiliary to financial intermediation (67) | Meuro |
| Real estate services (70) | Meuro |
| Renting services of machinery and equipment without operator and of personal and household goods (71) | Meuro |
| Research and development services (73) | Meuro |
| Other business services (74) | Meuro |
| Public administration and defence services; compulsory social security services (75) | Meuro |
| Education services (80) | Meuro |
| Health and social work services (85) | Meuro |
| Membership organisation services n.e.c. (91) | Meuro |
| Recreational; cultural and sporting services (92) | Meuro |
| Other services (93) | Meuro |
| Private households with employed persons (95) | Meuro |
| Extra-territorial organizations and bodies | Meuro |

| **List of products and services quantified in Meuro in EXIOBASE 3, but that correspond to products (e.g. motor vehicles), to services along the supply-chain of products (e.g. transmission and distribution of electricity, maintenance of motor vehicles) and to services quantifiable in physical units (e.g. transport services)** | Unit |
| --- | --- |
| Motor vehicles; trailers and semi-trailers (34) | Meuro |
| Other transport equipment (35) | Meuro |
| Secondary raw materials | Meuro |
| Transmission services of electricity | Meuro |
| Distribution and trade services of electricity | Meuro |
| Distribution services of gaseous fuels through mains | Meuro |
| Collected and purified water; distribution services of water (41) | Meuro |
| Construction work (45) | Meuro |
| Hotel and restaurant services (55) | Meuro |
| Railway transportation services | Meuro |
| Other land transportation services | Meuro |
| Sea and coastal water transportation services | Meuro |
| Inland water transportation services | Meuro |
| Air transport services (62) | Meuro |
| Sale; maintenance; repair of motor vehicles; motor vehicles parts; motorcycles; motor cycles parts and accessories | Meuro |
| Transportation services via pipelines | Meuro |
| Computer and related services (72) | Meuro |

| **EXIOBASE 3: list of products quantifiable in physical units (tonnes of product, TJ of electricity or tonnes of waste treated)** | Unit |
| --- | --- |
| Paddy rice | tonnes |
| Wheat | tonnes |
| Cereal grains nec | tonnes |
| Vegetables; fruit; nuts | tonnes |
| Oil seeds | tonnes |
| Sugar cane; sugar beet | tonnes |
| Plant-based fibers | tonnes |
| Crops nec | tonnes |
| Cattle | tonnes |
| Pigs | tonnes |
| Poultry | tonnes |
| Meat animals nec | tonnes |
| Animal products nec | tonnes |
| Raw milk | tonnes |
| Wool; silk-worm cocoons | tonnes |
| Manure (conventional treatment) | tonnes |
| Manure (biogas treatment) | tonnes |
| Products of forestry; logging and related services (02) | tonnes |
| Fish and other fishing products; services incidental of fishing (05) | tonnes |
| Anthracite | tonnes |
| Coking Coal | tonnes |
| Other Bituminous Coal | tonnes |
| Sub-Bituminous Coal | tonnes |
| Patent Fuel | tonnes |
| Lignite/Brown Coal | tonnes |
| BKB/Peat Briquettes | tonnes |
| Peat | tonnes |
| Crude petroleum and services related to crude oil extraction; excluding surveying | tonnes |
| Natural gas and services related to natural gas extraction; excluding surveying | tonnes |
| Natural Gas Liquids | tonnes |
| Other Hydrocarbons | tonnes |
| Uranium and thorium ores (12) | tonnes |
| Iron ores | tonnes |
| Copper ores and concentrates | tonnes |
| Nickel ores and concentrates | tonnes |
| Aluminium ores and concentrates | tonnes |
| Precious metal ores and concentrates | tonnes |
| Lead; zinc and tin ores and concentrates | tonnes |
| Other non-ferrous metal ores and concentrates | tonnes |
| Stone | tonnes |
| Sand and clay | tonnes |
| Chemical and fertilizer minerals; salt and other mining and quarrying products n.e.c. | tonnes |
| Products of meat cattle | tonnes |
| Products of meat pigs | tonnes |
| Products of meat poultry | tonnes |
| Meat products nec | tonnes |
| products of Vegetable oils and fats | tonnes |
| Dairy products | tonnes |
| Processed rice | tonnes |
| Sugar | tonnes |
| Food products nec | tonnes |
| Beverages | tonnes |
| Fish products | tonnes |
| Tobacco products (16) | tonnes |
| Textiles (17) | tonnes |
| Wearing apparel; furs (18) | tonnes |
| Leather and leather products (19) | tonnes |
| Wood and products of wood and cork (except furniture); articles of straw and plaiting materials (20) | tonnes |
| Wood material for treatment; Re-processing of secondary wood material into new wood material | tonnes |
| Pulp | tonnes |
| Secondary paper for treatment; Re-processing of secondary paper into new pulp | tonnes |
| Paper and paper products | tonnes |
| Printed matter and recorded media (22) | tonnes |
| Coke Oven Coke | tonnes |
| Gas Coke | tonnes |
| Coal Tar | tonnes |
| Motor Gasoline | tonnes |
| Aviation Gasoline | tonnes |
| Gasoline Type Jet Fuel | tonnes |
| Kerosene Type Jet Fuel | tonnes |
| Kerosene | tonnes |
| Gas/Diesel Oil | tonnes |
| Heavy Fuel Oil | tonnes |
| Refinery Gas | tonnes |
| Liquefied Petroleum Gases (LPG) | tonnes |
| Refinery Feedstocks | tonnes |
| Ethane | tonnes |
| Naphtha | tonnes |
| White Spirit & SBP | tonnes |
| Lubricants | tonnes |
| Bitumen | tonnes |
| Paraffin Waxes | tonnes |
| Petroleum Coke | tonnes |
| Non-specified Petroleum Products | tonnes |
| Nuclear fuel | tonnes |
| Plastics; basic | tonnes |
| Secondary plastic for treatment; Re-processing of secondary plastic into new plastic | tonnes |
| N-fertiliser | tonnes |
| P- and other fertiliser | tonnes |
| Chemicals nec | tonnes |
| Charcoal | tonnes |
| Additives/Blending Components | tonnes |
| Biogasoline | tonnes |
| Biodiesels | tonnes |
| Other Liquid Biofuels | tonnes |
| Rubber and plastic products (25) | tonnes |
| Glass and glass products | tonnes |
| Secondary glass for treatment; Re-processing of secondary glass into new glass | tonnes |
| Ceramic goods | tonnes |
| Bricks; tiles and construction products; in baked clay | tonnes |
| Cement; lime and plaster | tonnes |
| Ash for treatment; Re-processing of ash into clinker | tonnes |
| Other non-metallic mineral products | tonnes |
| Basic iron and steel and of ferro-alloys and first products thereof | tonnes |
| Secondary steel for treatment; Re-processing of secondary steel into new steel | tonnes |
| Precious metals | tonnes |
| Secondary preciuos metals for treatment; Re-processing of secondary preciuos metals into new preciuos metals | tonnes |
| Aluminium and aluminium products | tonnes |
| Secondary aluminium for treatment; Re-processing of secondary aluminium into new aluminium | tonnes |
| Lead; zinc and tin and products thereof | tonnes |
| Secondary lead for treatment; Re-processing of secondary lead into new lead | tonnes |
| Copper products | tonnes |
| Secondary copper for treatment; Re-processing of secondary copper into new copper | tonnes |
| Other non-ferrous metal products | tonnes |
| Secondary other non-ferrous metals for treatment; Re-processing of secondary other non-ferrous metals into new other non-ferrous metals | tonnes |
| Foundry work services | tonnes |
| Fabricated metal products; except machinery and equipment (28) | tonnes |
| Machinery and equipment n.e.c. (29) | tonnes |
| Office machinery and computers (30) | tonnes |
| Electrical machinery and apparatus n.e.c. (31) | tonnes |
| Radio; television and communication equipment and apparatus (32) | tonnes |
| Medical; precision and optical instruments; watches and clocks (33) | tonnes |
| Furniture; other manufactured goods n.e.c. (36) | tonnes |
| Bottles for treatment; Recycling of bottles by direct reuse | tonnes |
| Electricity by coal | TJ |
| Electricity by gas | TJ |
| Electricity by nuclear | TJ |
| Electricity by hydro | TJ |
| Electricity by wind | TJ |
| Electricity by petroleum and other oil derivatives | TJ |
| Electricity by biomass and waste | TJ |
| Electricity by solar photovoltaic | TJ |
| Electricity by solar thermal | TJ |
| Electricity by tide; wave; ocean | TJ |
| Electricity by Geothermal | TJ |
| Electricity nec | TJ |
| Coke oven gas | tonnes |
| Blast Furnace Gas | tonnes |
| Oxygen Steel Furnace Gas | tonnes |
| Gas Works Gas | tonnes |
| Biogas | tonnes |
| Steam and hot water supply services | TJ |
| Secondary construction material for treatment; Re-processing of secondary construction material into aggregates | tonnes |
| Food waste for treatment: incineration | tonnes |
| Paper waste for treatment: incineration | tonnes |
| Plastic waste for treatment: incineration | tonnes |
| Intert/metal waste for treatment: incineration | tonnes |
| Textiles waste for treatment: incineration | tonnes |
| Wood waste for treatment: incineration | tonnes |
| Oil/hazardous waste for treatment: incineration | tonnes |
| Food waste for treatment: biogasification and land application | tonnes |
| Paper waste for treatment: biogasification and land application | tonnes |
| Sewage sludge for treatment: biogasification and land application | tonnes |
| Food waste for treatment: composting and land application | tonnes |
| Paper and wood waste for treatment: composting and land application | tonnes |
| Food waste for treatment: waste water treatment | tonnes |
| Other waste for treatment: waste water treatment | tonnes |
| Food waste for treatment: landfill | tonnes |
| Paper for treatment: landfill | tonnes |
| Plastic waste for treatment: landfill | tonnes |
| Inert/metal/hazardous waste for treatment: landfill | tonnes |
| Textiles waste for treatment: landfill | tonnes |
| Wood waste for treatment: landfill | tonnes |

S 11. Calculation of the impacts associated with the use phase in the IO-CF

The use phase in the IO-CF is calculated in an approach consistent with that of the process-based LCA. It includes direct emissions, electricity consumption, wastewater treatment and fossil fuels, steam and hot water. The table below reports the elements relative to household consumption (products and services consumed, direct emissions or use) that are considered as part of (are allocated to) the use phase in the IO-CF.

| **General description** | **EXIOBASE nomenclature** |
| --- | --- |
| **Direct** | Direct emissions or use |
| **Electricity consumption** | Production of electricity by coal |
|  | Production of electricity by gas |
|  | Production of electricity by nuclear |
|  | Production of electricity by hydro |
|  | Production of electricity by wind |
|  | Production of electricity by petroleum and other oil derivatives |
|  | Production of electricity by biomass and waste |
|  | Production of electricity by solar photovoltaic |
|  | Production of electricity by solar thermal |
|  | Production of electricity by tide, wave, ocean |
|  | Production of electricity by Geothermal |
|  | Production of electricity nec |
|  | Transmission of electricity |
|  | Distribution and trade of electricity |
| **Wastewater treatment** | Waste water treatment, food |
|  | Waste water treatment, other |
| **Fossil fuels, steam and hot water** | Mining of coal and lignite; extraction of peat (10) |
|  | Extraction of crude petroleum and services related to crude oil extraction, excluding surveying |
|  | Extraction of natural gas and services related to natural gas extraction, excluding surveying |
|  | Extraction, liquefaction, and regasification of other petroleum and gaseous materials |
|  | Manufacture of coke oven products |
|  | Petroleum Refinery |
|  | Manufacture of gas; |
|  | Distribution of gaseous fuels through mains |
|  | Steam and hot water supply |

S 12. Hotspot analysis, by substance: focus on toxicity and ecotoxicity-related impacts

The analysis of the contribution by substance to toxicity-related impacts highlights that some of the substances that are of most relevance for the pLCA-CF are excluded from EXIOBASE 3, and accordingly from the IO-CF. In fact, in EXIOBASE 3 there are no emissions of pesticides, no emissions of metals to water and the only emission of metals to soil are emissions of zinc and lead. This difference mainly explains both the lower contribution of the food sector in the IO-CF (regarding HTOX-c, HTOX-nc and ECOTOX), and the lower impact of the IO-CF in absolute terms (regarding HTOX-c and ECOTOX; Table 1 in the article). In both approaches, the substance that contributes the most to the impact on HTOX-c is Chromium (Cr). However, in the case of the top-down approach, 80% of the impact is generated by the emission of Cr to air, whereas in the bottom-up approach Cr to air contributes only for 10% to the total impact, while the emissions of chromium to water, both as Cr and Cr VI, contribute to 40% and 30% respectively. The exclusion of chromium emissions to water from EXIOBASE 3 explains a major share of the lower absolute impact calculated with the top-down approach compared to the one calculated with the bottom-up (Table 1). In fact, when excluding the contribution of emissions of chromium to water, the impact calculated with the pLCA-CF is 1.98*10^4^ CTUh, which is closer to the one of the top-down without services (1.78*10^4^ CTUh), even if still slightly higher (11%).

Moreover, the impact on HTOX-nc is mostly generated by the emission of Pb to air (more than 40%) and Hg to air (around 40%) in the top-down approach. In the case of the bottom-up, similarly, emission of Hg to air contributes to around 40% of the impact; on the contrary, the contribution of emission of Pb to air is lower than in the top-down (10%) while the rest of the impact is generated by emissions of Zn, Hg, Pb and Cd to soil and by emission of As to air. Looking at the inventory, it emerges that the emission of Pb to air is lower in the pLCA-CF (2.46*10^6^ kg) than it is in the IO-CF (2.14*10^7^ kg) and the same is for Hg (9.33*10^4^ kg in the pLCA-CF and 2.50*10^5^ kg in the IO-CF). Those emissions come mainly from the cultivation of feed used for animal breeding in the bottom-up approach, whereas, in the environmental extensions of EXIOBASE 3, no emission of metals to soil is accounted for as stemming from the agricultural sector.

Similarly, the pLCA-CF identifies a longer list of substances contributing to ECOTOX, compared to the IO-CF, which is dominated by the emission of Zinc to air (51%) and Chromium to air (31%). Inventory values confirm the difference in the amount of emissions of Zn to air accounted for: 6.50*10^6^ kg in the pLCA-CF and 1.62*10^7^ kg in the IO-CF. On the contrary, the difference between the two approaches in terms of emission of Cr to air at the inventory stage is not as high as their contribution in relative terms: 3.67*10^6^ kg in the pLCA-CF and 7.91*10^6^ kg in the IO-CF. It has to be considered that some of the substances that contribute to the impact in the pLCA-CF are not accounted for in EXIOBASE 3 and accordingly in the IO-CF (e.g. Chromium and Chromium VI to water, Zn to water, Sb to air, Chlorpyriphos to soil and Folpet to soil). When the contribution of those substances is excluded also in the bottom-up, the impact is 1.09*10^12^ CTUe, which is still twice higher than the one calculated with the top-down (equal to 4.94*10^11^ CTUe, excluding services). This means that the different list of substances considered is a main driver for the difference between the two approaches (the factor of difference between pLCA-CF and IO-CF amounts to 14 when considering all substances in the pLCA-CF), but is not the only driver.
